# Light and dark cycles modify the expression of clock genes in the ovaries of *Aedes aegypti* in a noncircadian manner

**DOI:** 10.1371/journal.pone.0287237

**Published:** 2023-10-19

**Authors:** Leo Nava Piorsky Dominici Cruz, Rayane Teles-de-Freitas, Maria Eduarda Barreto Resck, Andresa Borges de Araujo Fonseca, Karine Pedreira Padilha, Luana Cristina Farnesi, Luciana Ordunha Araripe, Rafaela Vieira Bruno

**Affiliations:** 1 Laboratório de Biologia Molecular de Insetos, Instituto Oswaldo Cruz, Fundação Oswaldo Cruz, Rio de Janeiro- RJ, Brazil; 2 Instituto Nacional de Ciência e Tecnologia em Entomologia Molecular (INCT-EM), CNPq, Rio de Janeiro- RJ, Brazil; Karlsruhe Institute of Technology, GERMANY

## Abstract

Circadian oscillators (*i*.*e*., circadian clocks) are essential to producing the circadian rhythms observed in virtually all multicellular organisms. In arthropods, many rhythmic behaviors are generated by oscillations of the central pacemaker, specific groups of neurons of the protocerebrum in which the circadian oscillator molecular machinery is expressed and works; however, oscillators located in other tissues (*i*.*e*., peripheral clocks) could also contribute to certain rhythms, but are not well known in non-model organisms. Here, we investigated whether eight clock genes that likely constitute the *Aedes aegypti* clock are expressed in a circadian manner in the previtellogenic ovaries of this mosquito. Also, we asked if insemination by conspecific males would alter the expression profiles of these clock genes. We observed that the clock genes do not have a rhythmic expression profile in the ovaries of virgin (VF) or inseminated (IF) females, except for *period*, which showed a rhythmic expression profile in ovaries of IF kept in light and dark (LD) cycles, but not in constant darkness (DD). The mean expression of seven clock genes was affected by the insemination status (VF or IF) or the light condition (LD 12:12 or DD), among which five were affected solely by the light condition, one solely by the insemination status, and one by both factors. Our results suggest that a functional circadian clock is absent in the ovaries of *A*. *aegypti*. Still, their differential mean expression promoted by light conditions or insemination suggests roles other than circadian rhythms in this mosquito’s ovaries.

## Introduction

Endogenous circadian oscillators are the key to maintaining circadian rhythms (with a period of around 24 hours) in most organisms, including insects [1]. Such oscillators are based on the interaction of molecular components (*i*.*e*., genes and proteins), some of which receive exogenous inputs (*e*.*g*., environmental, social clues, etc.), keep circadian feedback loop interactions, and lead to outputs rhythms observed in those organisms, like foraging [2], sleep [3] and oviposition [4, 5].

Most information about endogenous insect oscillators is based on the model organism *Drosophila melanogaster*. Studies on *D*. *melanogaster* revealed that specific groups of neurons in the central nervous system (CNS) express the major players in the molecular machinery that run the positive and negative feedback loop interactions driving rhythmic outputs [6, 7]. This oscillatory mechanism in the CNS is conserved in almost all multicellular animals [8–11]; however, other tissues, like Malpighian tubules, prothoracic gland, and testis might also present cell groups that possess oscillatory mechanisms [12, 13].

In *D*. *melanogaster*, peripheral clocks in various tissues can be classified regarding their dependence on the rhythms produced by a central pacemaker, leading to different hierarchical levels of rhythm production. While clocks in some tissues (*e*.*g*., oenocytes) are entrained exclusively by the central pacemaker, others (*e*.*g*., the prothoracic glands) are entrained either by the central pacemaker or light inputs. However, most peripheral clocks, like the one in the Malpighian tubules, produce rhythms without participation from the central pacemaker [12, 13].

Studies in the peripheral clocks of non-model arthropods are still incipient. Yet, Kotwica et al. [2009] and Tobback et al. [2011] demonstrated that in *D*. *melanogaster* and *Locusta migratoria*, some clock genes are expressed in reproductive tissues and are essential in order for those organisms to fulfill their reproductive potential [14, 15]. However, few studies investigated the temporal expression profiles of clock genes and the existence of peripheral oscillators in reproductive tissues [16, 17]. Such investigations are essential, as oscillators located in gonadal tissues could be involved in reproductive outputs like sperm liberation and oviposition, which indeed show rhythmic patterns [4, 18].

Our group has been investigating the behavioral rhythms and the circadian profile of clock genes in the mosquito *Aedes aegypti* (L.), a hematophagous insect that is a competent vector of several causative pathogens of many human diseases [19, 20]. *Aedes aegypti* shows evident rhythmic outputs in many phenotypes, including reproductive behaviors like oviposition [21, 22]. Whether those outputs are generated exclusively by the central clock or aided by peripheral oscillators is still unknown. On the other hand, discrete events in the reproductive life story, like insemination and blood-feeding, modify deeply the behavior and gene expression of females [23–25], which could result from a modified oscillation of the endogenous clocks.

Despite such aspects, the existence of peripheral clocks in reproductive tissues of *A*. *aegypti* is still unknown, even though the circadian oscillation of the clock genes in heads and headless bodies of this mosquito have been described [26–29]. Therefore, here we investigated the temporal expression profiles of eight insect clock genes in the ovaries of *A*. *aegypti* previously analyzed in the central clock of this mosquito: *period* (*per*), *timeless* (*tim*), *Clock* (*Clk*), *cycle* (*cyc*), *vrille* (*vri*), *Par-domain-protein-1ε* (*Pdp1ε*), *cryptochrome 1* (*cry1*) and *cryptochrome 2* (*cry2*). Furthermore, we aimed to know whether insemination- the first major reproductive event in females in the previtellogenic state- could modify the temporal expression profiles that we investigated.

## Materials and methods

### Mosquito rearing and experimental groups separation

We studied *A*. *aegypti* mosquitoes of the Paea strain from Tahiti, French Polynesia [30]. The eggs, obtained from a colony maintained in the Laboratório de Mosquitos Transmissores de Hematozoários of the Instituto Oswaldo Cruz (LATHEMA-IOC) since 2003, were hatched in plastic trays containing 1.5 L of Milli-RO^TM^ water. Immature stages were reared from the first instar larvae (L1) to pupae by feeding on 1g of yeast pellets (Vitalab^®^, Brazil) and maintained in B.O.D. (Biochemical Oxygen Demand) incubators set to 25°C ±1°C and a photoperiod of 12 hours of light and 12 hours of darkness (LD 12:12). Before the emergence of adults, we visually separated the pupae in males and females: larger pupae were identified as females and smaller pupae as males. Separation by size had 100% accuracy, as verified after adults emerged. Female pupae were split into two groups for the experiments: virgin females (VF)- kept in mosquito cages without males- and inseminated females (IF)—kept in cages with males (2:1 male: female) for four days to be inseminated.

On the fourth day post-emergence, VF and IF were removed from the cages, placed in Falcon® tubes, and entrained for three more days at 25± 1° C and LD 12:12. On the following day (4^th^ day of the experiment), females were sampled every four hours for 24 hours, either in LD 12:12 or in DD (constant darkness), to verify the clock genes expression in free running at *Zeitgeber* Times (ZTs) or Circadian Times (CTs) 1, 5, 9, 13, 17 and 21. Sampled females were frozen in dry ice and immediately stored at -80° C until tissue dissections. Our results were based on three to four independent experiments in each photoperiod.

We dissected both ovaries from the sampled females in sterile phosphate-buffered saline (PBS) over an ice-cold metal plate under a stereomicroscope. For each ZT/CT, a pool of 15 pairs of ovaries was stored in a microtube containing 200μl of TRIzol^TM^ (ThermoFisher Scientific^©^) solution and kept at -80° C until RNA extraction. The three spermathecae of virgin and inseminated females were dissected in PBS on a glass slide and visualized under a microscope to determine the insemination status.

### Molecular assays

We extracted the total RNA from the female pools in line with the TRIzol^TM^ manufacturer protocol, followed by 2.5M Lithium Chloride (LiCl) purification, according to Gentile et al. [26]. Total RNA concentration was measured by the Qubit 3.0 Fluorometric Quantitation method (ThermoFisher Scientific©), according to the manufacturer’s instructions, and then diluted to 5 ng/μl. A volume of 4 μl of diluted RNA was used for the cDNA synthesis by TaqMan® Reverse Transcription Reagents (Invitrogen^TM^).

In the pacemaker (or central clock) of insects, interactions of the clock proteins activate or repress the expression pattern of their own genes, promoting a well-defined 24-hour-gene expression profile [31]. Therefore, we analyzed, in the ovaries of *A*. *aegypti*, the temporal expression profiles of eight clock genes previously characterized in heads (where the central clock of insects is located) of *A*. *aegypti* females by Gentile et al. [26] and available at VectorBase- a part of the VEuPathDB: the eukaryotic pathogen, vector, and host bioinformatics resource center [32]: *per* (available at VectorBase under the accession number AAEL008141), *tim* (AAEL019461), *Clk* (AAEL022593), *cyc* (AAEL002049), *vri* (AAEL011371), *Pdp1ε* (AAEL005255), *cry1* (AAEL004146) and *cry2* (AAEL011967).

The relative abundances of mRNA for samples from each time point were accessed by quantitative Polymerase Chain Reaction (qPCR), performed with Power SYBR Green Master Mix (Applied Biosystems©) and 10 μM of each primer (sequences described by Gentile et al.[26]), at the StepOnePlus™ Real-Time PCR System (Applied Biosystems©). Expression values were obtained by the 2^–ΔΔCt^ method [33] using the *rp49* (AAEL003396) ribosomal gene as the endogenous control with the primers described by Gentile et al. [34].

### Data analysis

To access the relative expression values in temporal profiles, we normalized the ΔCt values of the six-time points of a given experimental condition (*e*.*g*., VF in LD 12:12) to the higher ΔCt value (*i*.*e*., the lower expression value) in that series, according to Gentile et al. [26]. We compared the expression level among ZTs/CTs by one-way Analysis of Variance (ANOVA), applied to the normalized logarithmic 2^–ΔΔCt^ values, followed by multiple comparisons by Tukey post-hoc test. These values were also analyzed by the CircWave v 1.4 software, which verifies whether the data are distributed periodically by comparing them with Fourier curves [35]. We considered the expression profile as rhythmic if both one-way ANOVA and CircWave pointed to a significant difference (*i*.*e*., p ≤ 0.05) between ZTs/CTs. The mean relative abundance values and their standard error values (SE) were used to build graphs of the gene expression profiles.

To access the isolated or the synergic contribution from physiological status (*i*.*e*., VF and IF) and light conditions (LD 12:12 and DD), we considered the overall expression values of all time points in each experimental state. Then, we normalized those values to the higher ΔCt value common to all conditions. We compared the mean 2^–ΔΔCt^ values of each experimental condition through two-way ANOVA, followed by Bonferroni’s post-hoc test. Both one and two-way ANOVAs were performed using GraphPad Prism v 5.0 software (GraphPad Software Inc.- San Diego, CA, EUA).

## Results and discussion

**Fig 1:**
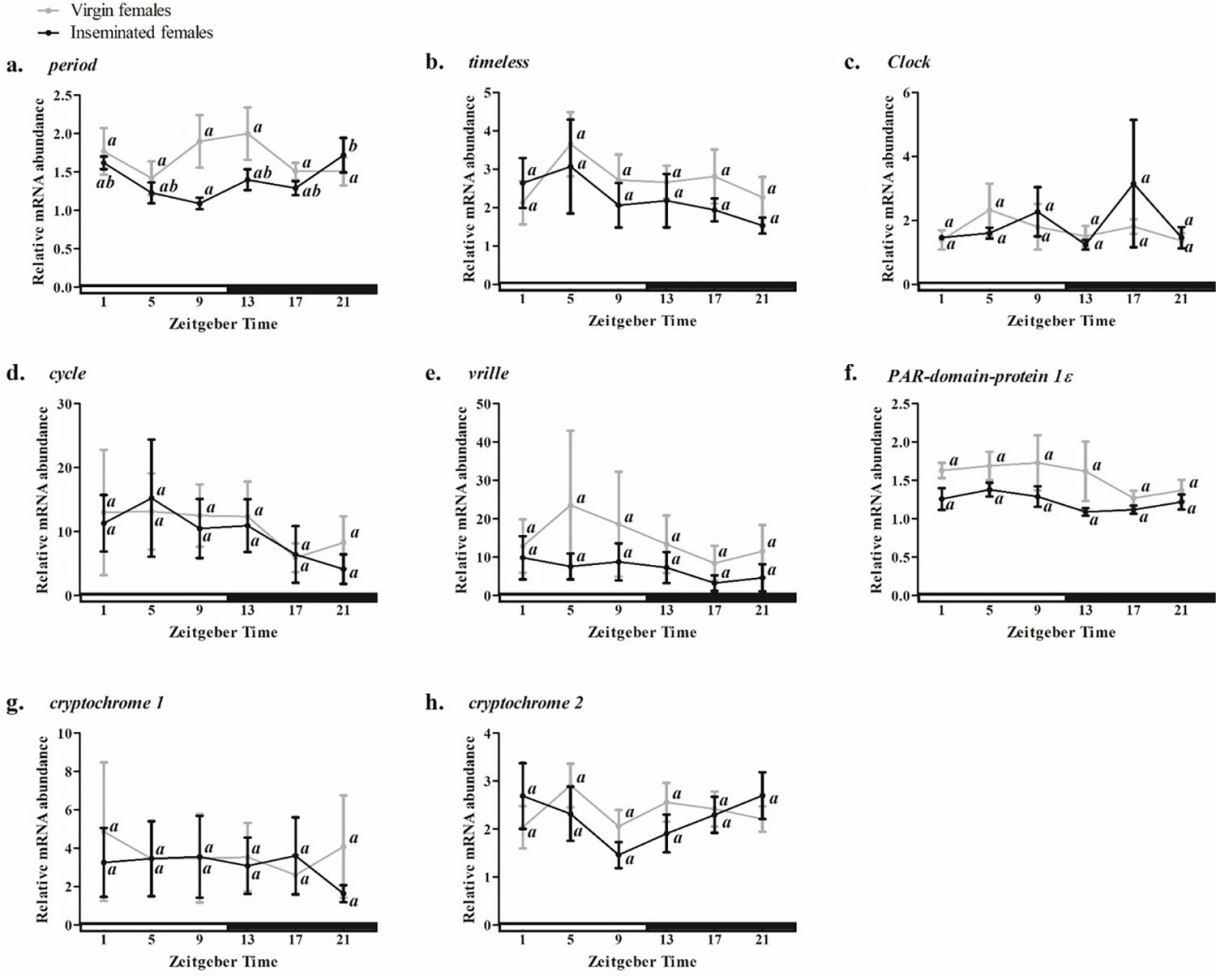
Temporal profile of relative expression of clock genes in ovaries of virgin and inseminated *Aedes aegypti* females kept in LD 12:12. The graph shows the expression of *per* (a), *tim* (b), *Clk* (c), *cyc* (d), *vri* (e), *Pdp1ε* (f), *cry1* (g) and *cry2* (h). The vertical axis indicates the relative abundance of messenger RNA and the horizontal axis, the Zeitgeber Time (ZT). White bars indicate the light phase of the photoperiod, and black bears, the dark phase. ZT1 represents one hour after lights turn on and ZT13 one hour after lights turn off. Vertical bars represent standard errors (SEM). The graphs were obtained by the average of three to four independent experiments. Data analysis was performed using the one-way Analysis of Variance (one-way ANOVA). Different lowercase letters indicate that the values of the respective time points differ significantly from each other in the same profile, according to Tukey post-hoc test (p < 0.05).

We used the one-way ANOVA to access potential differences among the logarithmic 2^–ΔΔCt^ values of time points of each studied clock gene. Concurrently, we evaluated whether those expression values would be distributed in an oscillatory model through a cosinor-based analysis in the software CircWave v1.4. The one-way ANOVA results showed no differences among time points for any of the clock genes in the ovaries of virgin females (VF) kept in a photoperiod of 12 hours of lightness and 12 hours of darkness (LD 12:12) (Fig 1 and Table 1), supported by CircWave analysis, except for *Pdp1ε* (*p* = 0.0421; R^2^ = 0.26; Table 1).

**Table 1:** Analysis of variance (one-way ANOVA) comparing the expression in six time points and CircWave values of daily expression of clock genes in the ovaries of virgin (VF) and inseminated (IF) *Aedes aegypti* females submitted to a LD12:12 photoperiod.

Gene	one-way ANOVA		CircWave	
	*F*	*p*	*p*	R^2^
VF				
*period*	*F*_*5*,*18*_ = 1.640	0.2002 (NS)	(NS)	0
*timeless*	*F*_*5*,*18*_ = 0.7268	0.6123 (NS)	(NS)	0
*Clock*	*F*_*5*,*12*_ = 0.5144	0.7607 (NS)	(NS)	0
*cycle*	*F*_*5*,*18*_ = 0.6929	0.6354 (NS)	(NS)	0
*vrille*	*F*_*5*,*18*_ = 0.8297	0.5452 (NS)	(NS)	0
*Par-domain-protein 1ε*	*F*_*5*,*18*_ = 1.454	0.2532 (NS)	0.0421 (*)	0.26
*cryptochrome 1*	*F*_*5*,*12*_ = 0.3545	0.8697 (NS)	(NS)	0
*cryptochrome 2*	*F*_*5*,*18*_ = 1.066	0.4111 (NS)	(NS)	0
IF				
***period***	***F***_***5*,*18***_ **= 3.426**	**0.0238** (*)	**0.0109** (*)	**0.35**
*timeless*	*F*_*5*,*18*_ = 0.5956	0.7037 (NS)	(NS)	0
*Clock*	*F*_*5*,*12*_ = 0.5186	0.7578 (NS)	(NS)	0
*cycle*	*F*_*5*,*18*_ = 0.6241	0.6835 (NS)	(NS)	0
*vrille*	*F*_*5*,*18*_ = 0.4219	0.8274 (NS)	(NS)	0
*Par-domain-protein 1ε*	*F*_*5*,*18*_ = 1.128	0.3810 (NS)	(NS)	0
*cryptochrome 1*	*F*_*5*,*12*_ = 0.1338	0.9814 (NS)	(NS)	0
*cryptochrome 2*	*F*_*5*,*18*_ = 0.9767	0.4584 (NS)	(NS)	0

NS: non-significant difference (p > 0.05)

*: significant difference (p < 0.05)

Most of the clock genes in the ovaries of inseminated females (IF) kept in LD 12:12 showed an arrhythmic pattern of expression, as pointed by one-way ANOVA and CircWave results (Table 1), except for *per*, whose values not only showed differences throughout the temporal profile but also adjusted in the oscillatory model of the CircWave (*p* = 0.0109; R^2^ = 0.35; Table 1, bold values). The oscillation in *per* showed a trough at ZT 9 (light phase), which differs significantly from the highest expression value at ZT 21 (dark phase), as pointed out by Tukey’s post-hoc test (Fig 1), although this decrease was slighter than that seen in the central clock [26]. Since this pattern did not remain in DD, we cannot consider this a circadian rhythm, but a masking effect caused by light. This non-circadian expression pattern of *per*, different from the rhythmic expression observed in heads [26], suggests that PER is not a component of clock machinery in the ovaries of *A*. *aegypti* but may have other roles, as emphasized by Rush et al. [36] after observing the constitutive cytoplasmic localization of PER in the ovaries of *D*. *melanogaster*.

**Fig 2:**
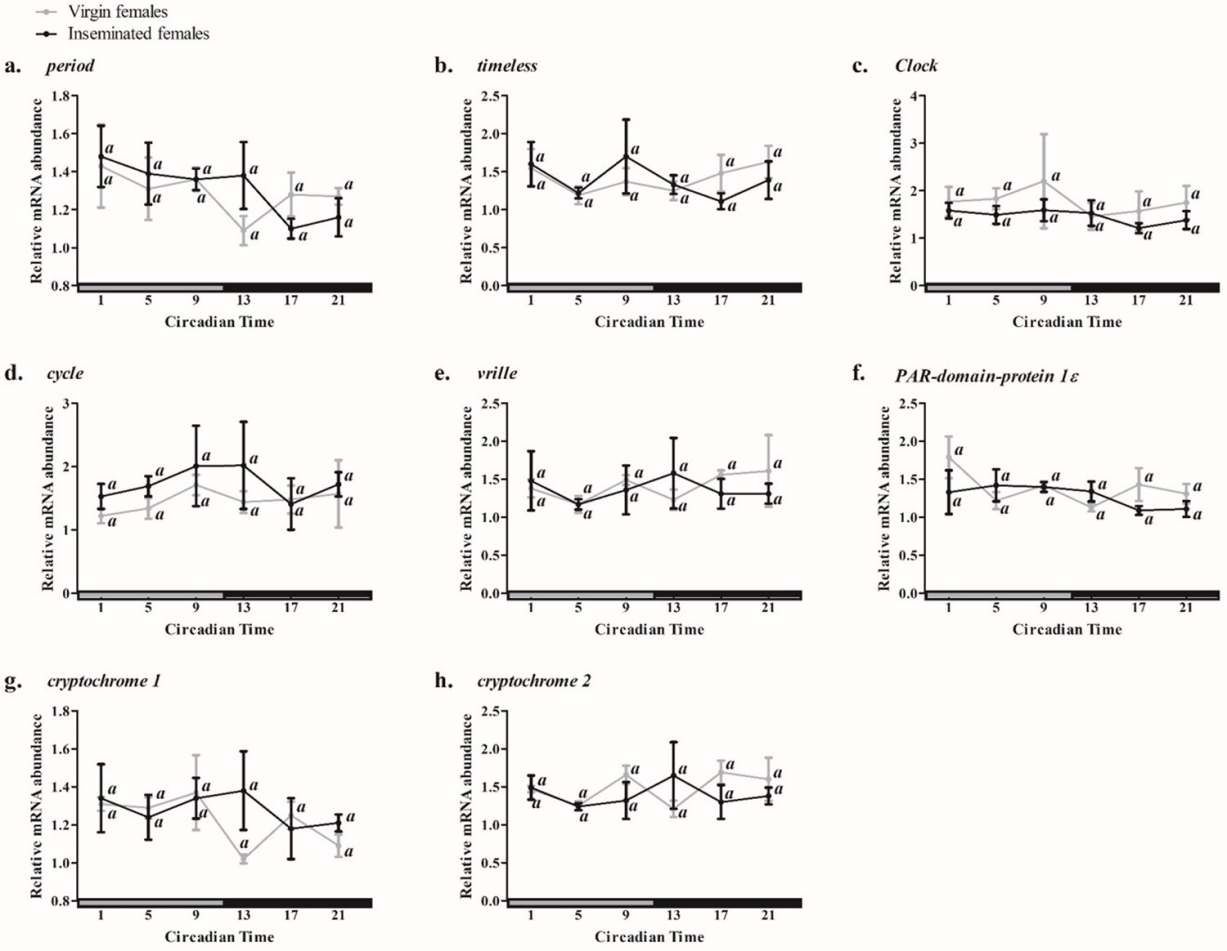
Temporal profile of relative expression of clock genes in ovaries of virgin and inseminated *Aedes aegypti* females kept in DD. The graph shows the expression of per (a), tim (b), Clk (c), cyc (d), vri (e), Pdp1ε (f), cry1 (g) and cry2 (h). The vertical axis indicates the relative abundance of messenger RNA and the horizontal axis, the Circadian Time (CT). Grey bars indicate the subjective day and black bars, the subjective night. CT1 represents one hour after the beginning of subjective day and CT13, one hour after the beginning of subjective night. Vertical bars represent standard error (SEM). The graphs were obtained by the average of three independent experiments. Data analysis was performed using the one-way ANOVA. Different lowercase letters indicate that the values of the respective time points differ significantly from each other in the same profile, according to Tukey’s post-hoc test (p < 0.05).

To confirm whether the temporal expression profiles observed in LD were truly circadian and not a masking effect caused by light, we submitted VF and IF to a period of 24 hours in constant darkness (DD); therefore, we could observe whether those profiles could persist or not in free running (*i*.*e*., persisting in the absence of daily environmental cycles, like LD). In the ovaries of VF kept in DD, one-way ANOVA did not show differences throughout the temporal expression profiles of clock genes, and none of them fit in the CircWave oscillatory model (Fig 2 and Table 2). No significant differences were found throughout the profiles of clock genes in the ovaries of IF kept in DD (Table 2 and Fig 2), even though the temporal expression values of *Pdp1ε* did fit in the CircWave model (R^2^ = 0.3351; Table 2).

**Table 2:** Analysis of variance (one-way ANOVA) comparing the expression in six time points and CircWave values of circadian expression of clock genes in the ovaries of virgin (VF) and inseminated (IF) *Aedes aegypti* females kept in constant darkness (DD).

Gene	one-way ANOVA		CircWave	
	*F*	*p*	*P*	R^2^
VF				
*period*	*F*_*5*,*12*_ = 1.022	0.4473 (NS)	(NS)	0
*timeless*	*F*_*5*,*12*_ = 1.097	0.4112 (NS)	(NS)	0
*Clock*	*F*_*5*,*12*_ = 0.7473	0.6034 (NS)	(NS)	0
*cycle*	*F*_*5*,*12*_ = 0.5910	0.7074 (NS)	(NS)	0
*vrille*	*F*_*5*,*12*_ = 0.9527	0.4829 (NS)	(NS)	0
*Par-domain-protein 1ε*	*F*_*5*,*12*_ = 2.349	0.1047 (NS)	(NS)	0
*cryptochrome 1*	*F*_*5*,*12*_ = 2.632	0.0787 (NS)	(NS)	0
*cryptochrome 2*	*F*_*5*,*12*_ = 1.487	0.2649 (NS)	(NS)	0
IF				
*period*	*F*_*5*,*12*_ = 1.361	0.3053 (NS)	(NS)	0
*timeless*	*F*_*5*,*12*_ = 0.6678	0.6553 (NS)	(NS)	0
*Clock*	*F*_*5*,*12*_ = 0.5557	0.7319 (NS)	(NS)	0
*cycle*	*F*_*5*,*12*_ = 0.2961	0.9059 (NS)	(NS)	0
*vrille*	*F*_*5*,*12*_ = 0.1392	0.9797 (NS)	(NS)	0
*Par-domain-protein 1ε*	*F*_*5*,*12*_ = 1.351	0.3086 (NS)	0.0468 (*)	0.3351
*cryptochrome 1*	*F*_*5*,*12*_ = 0.3391	0.8796 (NS)	(NS)	0
*cryptochrome 2*	*F*_*5*,*12*_ = 0.3452	0.8757 (NS)	(NS)	0

NS: non-significant difference (p > 0.05)

*: significant difference (p < 0.05)

Those results arrhythmic expression patterns agreed with those of *period* in the ovaries of *Drosophila melanogaster* [37, 38]. On the other hand, in the ovaries of the shrimp *Macrobrachium nipponense* in or out of the breeding season, *per* and *tim* exhibits expression values that vary among time points throughout 24 hours, suggesting a rhythmic expression profile [16]. Moreover, in the ovaries of mice, clock genes have a circadian expression in specific cell types, like the epithelial granulosa cells [39], suggesting that the arrhythmic expression of clock genes in ovaries is not widespread in animals. Studies on other insects are necessary to investigate whether this is a characteristic of all insect species or only some groups.

In the ovaries of *D*. *melanogaster*, the arrhythmic profiles of some clock genes are partially explained by the absence of expression of *cryptochrome* in those organs, since the ectopic expression of *cry* in the ovaries of this fly induces degradation of TIM and alters the cellular abundance of PER [36]. The sequestration and inducement of TIM to degradation via proteasome by CRY is an important phenomenon that occurs in the central pacemaker of *D*. *melanogaster*- and even in cell cultures of monarch butterflies- and is essential to entrain the clock during the light phase [40, 41]. However, this might not be the case with the ovaries of *A*. *aegypti*, as we demonstrated that *cry1*, the ortholog of *D*. *melanogaster cry*, was expressed at all time points in the ovaries of this mosquito.

Other components, like the kinase DOUBLETIME (DBT), are associated with the circadian change in cellular localization of PER in the ovaries of *D*. *melanogaster* (*i*.*e*., whether PER abundance is predominant in the cytoplasm or the nucleus) in the vitellogenic stage [42]. Since we evaluated gene expression only in previtellogenic ovaries of *A*. *aegypti*, it is possible that components like DBT were not expressed and hence were not contributing to the rhythmic behavior of clock components and, therefore, the rhythmic expression of the clock genes. However, preliminary data from our group showed that at least *tim*, *vri*, and *cry2* do not show rhythmic expression pattern along with an LD 12:12 photoperiod in blood-fed females when their follicles enter the vittelogenic stage (S1 Table and S1 Fig), suggesting that the expression of these genes do not show rhythmicity in the ovaries at any moment.

Fonseca et al. [43] showed that in the embryonic cell line, Aag2- derived from *A*. *aegypti*-most clock genes have not rhythmic expression profiles, probably due to the absence of central control of the circadian clock—a role played by the brain—in the cultured cells. It could also be the case when ovaries are studied in isolation from the brain, even though clock genes expression has significant rhythmicity when the headless bodies of *Aedes aegypti*, containing a variety of tissues, are studied [27].

The arrhythmicity of the clock genes expression in the ovaries of *A*. *aegypti* could also result from the heterogeneity of the number of follicles with different nutritional statuses. The ovaries of *A*. *aegypti* are dynamic organs. The fate of their follicles depends mainly on the previtellogenic feeding: females fed in a low concentration (3%) sucrose diet have more reabsorbed follicles during the vitellogenic stage in comparison to females fed on high concentrated (20%) sucrose diets and treated with juvenile hormone III (JH III) [44]. Moreover, the arrhythmic expression profiles of clock genes in the ovaries could be caused by the heterogeneity of the cell types in those organs (e.g., oocytes, follicular cells) since we could not dissect every cellular type in this study.

To understand whether the insemination status, light condition, or an interaction between them could affect the mean expression of clock genes, we compared, through Analysis of Variance (two-way ANOVA), their mean expression values in each experimental condition: VF in LD 12:12, VF in DD, IF in LD 12:12, and IF in DD. Our results showed that the mean expression of most of the studied clock genes- except by *tim*- were affected either by the insemination status or the light condition (Table 3). The lack of difference in *tim* mean expression among experimental conditions supports the findings of Teles-de-Freitas et al. [45] that the expression of this gene in the heads of *A*. *aegypti* females is only entrained by temperature cycles in phase with photoperiod.

**Table 3:** Analysis of variance (two-way ANOVA) comparing the mean expression values of the studied clock genes regarding the isolated and synergic effects of insemination status (VF and IF) and light condition (LD 12: 12 and DD).

Gene	Factors					
	Insemination		Light condition		Interaction	
	*F*_*1*,*20*_	*P*	*F*_*1*,*20*_	*p*	*F*_*1*,*20*_	*p*
*period*	1.653	0.2132	**35.38**	**<0.0001**	2.694	0.1164
*timeless*	2.684	0.1170	2.479	0.1311	1.574	0.2241
*Clock*	0.8809	0.3592	**6.264**	**0.0211**	2.060	0.1666
*Cycle*	0.6356	0.4347	**12.07**	**0.0024**	0.8867	0.3576
*Vrille*	**8.531**	**0.0085**	**4.512**	**0.0463**	**5.056**	**0.0360**
*PAR-domain-protein 1ε*	**9.382**	**0.0061**	0.7797	0.3877	1.981	0.1747
*cryptochrome 1*	0.002	0.9645	**145.0**	**<0.0001**	1.567	0.2250
*cryptochrome 2*	1.860	0.1877	**60.76**	**<0.0001**	0.03121	0.8615

The group of Virgin Females kept in LD 12:12 condition was used as the control. Significant values are highlighted in bold.

While investigating the effects of photoperiod and temperature cycles in *A*. *aegypti*, Teles-de-Freitas et al. [27] also showed that the expression of *tim*, *cyc*, and *cry2* in headless bodies (*i*.*e*., in putative peripheral clocks) of *A*. *aegypti* females become rhythmic only if those two zeitgebers are in phase with each other. Therefore, the investigation of clock genes expression in ovaries under temperature cycles coupled or not with photoperiod is a pertinent topic. Indeed, temperature modulates the oogenesis in *D*. *melanogaster* [46], suggesting this modulation also could affect the expression of the clock genes in the ovaries of *A*. *aegypti*.

**Fig 3:**
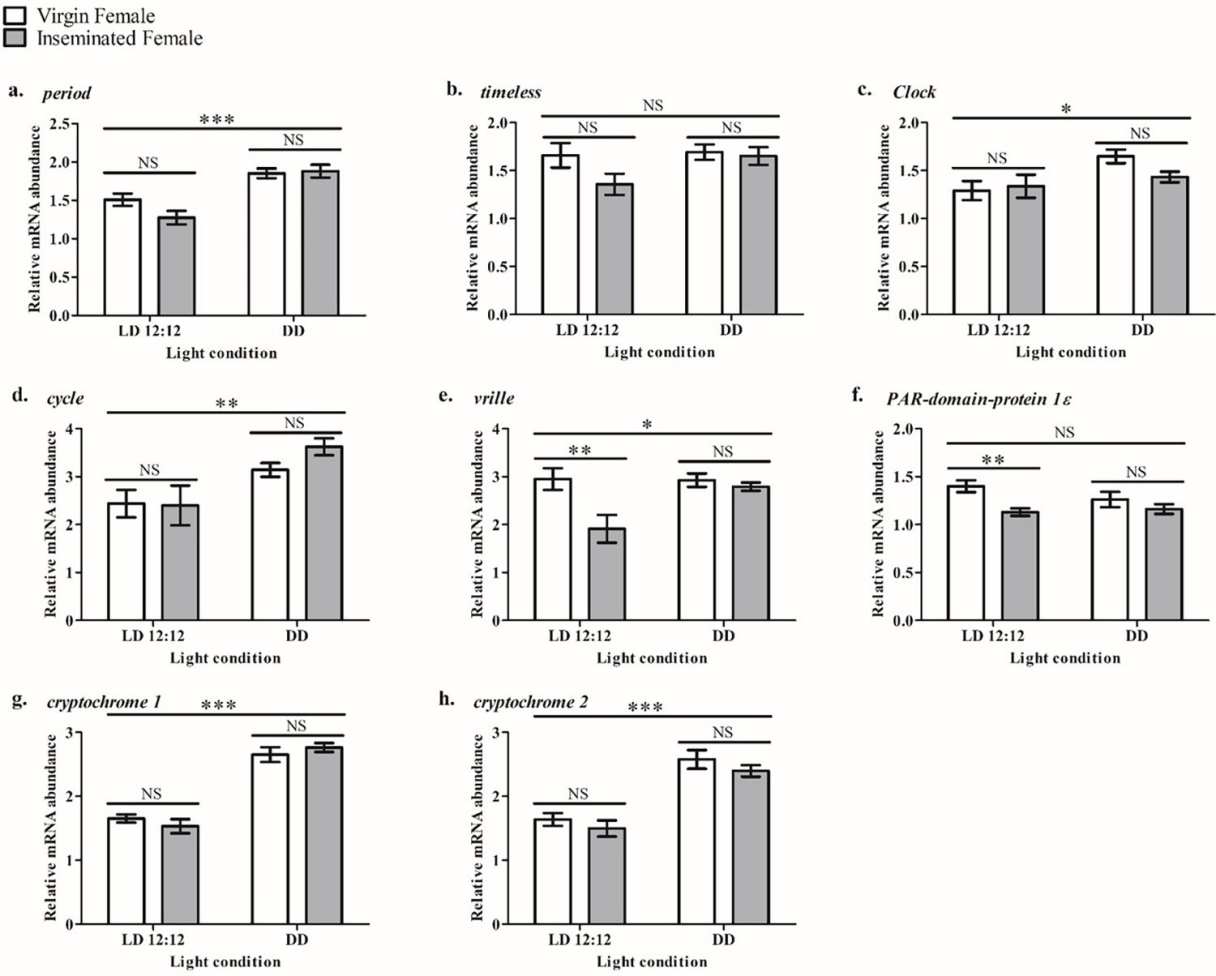
Graphic presentation of the two-way ANOVA comparing the mean expression values of the clock genes in ovaries of virgin and inseminated *Aedes aegypti* females kept in LD 12:12 and in DD. The graph shows the mean expression of *per* (a), *tim* (b), *Clk* (c), *cyc* (d), *vri* (e), *Pdp1ε* (f), *cry1* (g) and *cry2* (h). The vertical axis indicates the relative abundance of messenger RNA and the horizontal axis, the light condition (LD 12:12 or DD). The group of virgin females kept in LD 12:12 was used as the control. Virgin females are represented by white-filled bars, and inseminated females, by grey bars. Standard error (SEM) bars are presented above and below the mean. The graphs were obtained from the sum of expression values of all time points (ZTs or CTs) of each condition. Symbols and their meanings according to Bonferroni’s post-hoc test: NS (non-significant difference; p > 0.05); * (significant difference; p < 0.05); ** (significant difference; p < 0.01); *** (significant difference; p < 0.001).

Among the genes with differential mean expression, *per*, *Clk*, *cyc*, *cry1*, and *cry2* were affected exclusively by the light condition, with higher mean relative expression values in females kept in DD, with no effect from the insemination status, as pointed by Bonferroni’s post-hoc test (Fig 3). The insemination status had a more significant contribution (p < 0.01) than the light condition (p < 0.05), with a significant difference between experimental groups kept in LD 12:12 only (Fig 3).

The higher mean expression of *per*, *Clk*, *cyc*, *cry1*, and *cry2* observed in DD condition suggests that the presence of light in LD 12:12 reduces the expression of those genes in a non-circadian manner. This reduction had no participation of the insemination status (Table 3), supporting the data of Gentile et al. [47] that insemination status is not an essential factor in altering the expression of *per*, *tim*, *Clk*, and *cyc* neither in the heads nor in the bodies of *A*. *aegypti* females. As the number of eggs laid by *A*. *aegypti* females is also reduced during the light phase of LD cycles [22], the reduction of those clock genes’ mean expression caused by light would be an indication that they could participate in the egg-laying process of this mosquito, even that more studies are necessary to demonstrate this association.

The light condition similarly altered the mean expressions of *per*, *cry1*, and *cry2* (Table 3 and Fig 3). In heads of the moth *Spodoptera litura*, the expression of *cry1* is higher in the photophase than in the scotophase. The expression of *cry2* is higher in the scotophase in females and virgin males, leading the authors to argue for the role of CRY1 as a clock resetting component and CRY2 as a clock repressor component [48]. However, our data show that both *cry1* and *cry2* mean expressions are higher in DD condition (*i*.*e*., in the absence of light) than in LD 12:12, suggesting that those genes (and, by extension, *per*) have possibly redundant non-circadian roles in the ovaries of *A*. *aegypti*. As demonstrated by the role of CRY as a circadian repressor in peripheral clocks of *D*. *melanogaster*, but not in clock neurons [49], the same clock component may have different roles in different tissues; therefore, the expression pattern of their clock genes would also vary among tissues.

On the other hand, the mean expression of *Pdp1ε* was exclusively influenced by the insemination status, but only in LD 12:12 females, while the mean expression of *vri* was affected by both the insemination status and light condition, with a significant interaction between the factors (Table 3 and Fig 3). Even though the roles of *vri* and *Pdp1ε* in ovaries of *A*. *aegypti* are unknown, both genes are important for the embryogenesis process and growth of insects [50, 51]. Intriguingly, *vri* and *Pdp1ε* codify basic-region leucine zipper (bZIP) transcription factors, a large protein family that participates in plants’ development and whose genes are mostly strongly modulated by light [52]. Therefore, our data suggest that, unlike plants, the expression patterns of *vri* and *Pdp1ε* are affected not by light but by other factors, like insemination status.

## Conclusions

We herein demonstrated that the expression pattern of clock genes in the ovaries of *A*. *aegypti* is different from their pattern in heads [26, 45], which suggests that any rhythmic profile of the phenotypes seen in the ovaries of this mosquito would not be driven by circadian rhythms produced by the central pacemaker. Also, since Teles-de-Freitas et al. [27] demonstrated that *per* has a circadian expression in headless bodies of *A*. *aegypti* females, our results in ovaries suggest that specific tissues in the bodies of this mosquito have different hierarchies of control by the central pacemaker, as observed in *D*. *melanogaster* [12].

Regardless of the expression profile of clock genes in the ovaries, those genes are essential to keeping the optimal reproductive parameters of several arthropods [15, 53, 54], although few studies have decoupled the role of clock genes in peripheral tissues from the central clock (*e*.*g*. Kotwica et al. [14]). The pleiotropic roles of clock genes in *A*. *aegypti* ovarian development and its reproduction aspects remain to be explored.

## Supporting information

S1 Fig Temporal profile of relative expression of the clock genes in ovaries of inseminated and blood fed *Aedes aegypti* females kept in LD 12:12. The graph shows the expression of *timeless* (a), *vrille* (b), and *cryptochrome* 2 (c). The vertical axis indicates the relative abundance of messenger RNA and the horizontal axis, the Zeitgeber Time (ZT). White bars indicate the light phase of the photoperiod, and black bars, the dark phase. ZT3 represents three hours after lights-on and ZT15 three hours after lights-off. Vertical bars represent standard error (SEM). The graphs were obtained by the average of three independent experiments. Data analysis was performed using the one-way Analysis of Variance (ANOVA). The same lowercase letters indicate that the values of the respective time points did not differ significantly from each other in the profile, according to the Tukey test (p > 0.05).
(TIF)

S1 Table Analysis of variance (one-way ANOVA) comparing the expression in six time points and CircWave values of daily expression of clock genes in the ovaries of inseminated and blood fed *Aedes aegypti* females submitted to a LD12:12 photoperiod. (PDF)
