# Supplementary figures and images for: Light and dark cycles modify the expression of clock genes in the ovaries of Aedes aegypti in a noncircadian manner
Source: PLoS One. 2023 Oct 19;18(10):e0287237. doi: 10.1371/journal.pone.0287237 (PMC10586701; doi:10.1371/journal.pone.0287237)

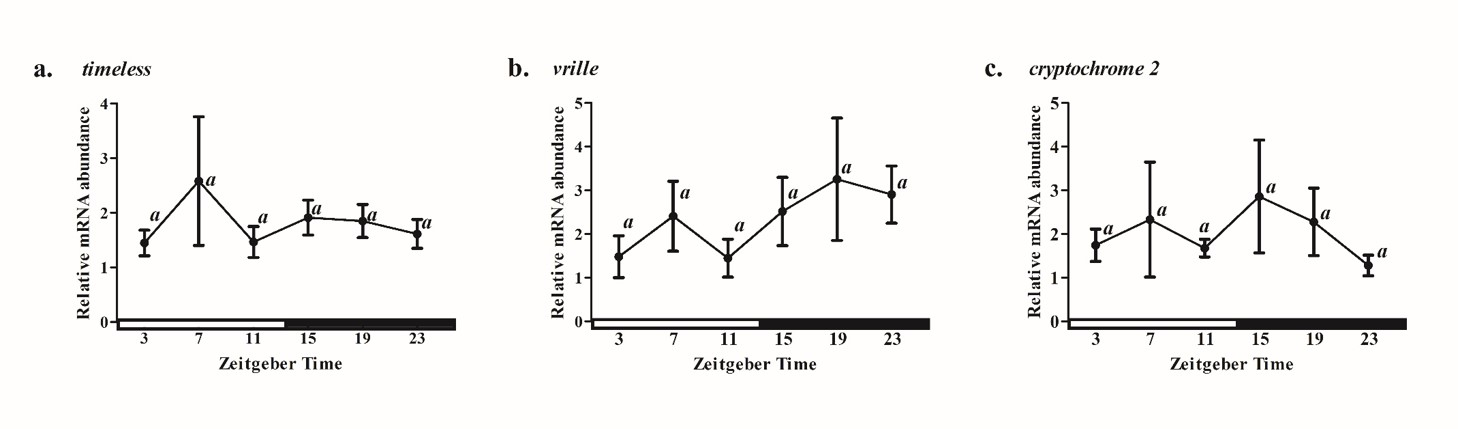

Supplement: S1 Fig — The graph shows the expression of timeless (a), vrille (b), and cryptochrome 2 (c). The vertical axis indicates the relative abundance of messenger RNA and the horizontal axis, the Zeitgeber Time (ZT). White bars indicate the light phase of the photoperiod, and black bars, the dark phase. ZT3 represents three hours after lights-on and ZT15 three hours after lights-off. Vertical bars represent standard error (SEM). The graphs were obtained by the average of three independent experiments. Data analysis was performed using the one-way Analysis of Variance (ANOVA). The same lowercase letters indicate that the values of the respective time points did not differ significantly from each other in the profile, according to the Tukey test (p > 0.05). (TIF) [file pone.0287237.s001.tif]
