# Supplementary material for: Light and dark cycles modify the expression of clock genes in the ovaries of Aedes aegypti in a noncircadian manner
Source: PLoS One. 2023 Oct 19;18(10):e0287237. doi: 10.1371/journal.pone.0287237 (PMC10586701; doi:10.1371/journal.pone.0287237)
Supplement: S1 Table — (PDF) [file pone.0287237.s002.pdf]

Gene	One-Way ANOVA		CircWave	
	F	p	p	R^2
timeless	$F_{5,18}$ = 0.4217	0.8249 (NS)	(NS)	0
vrille	$F_{5,18}$ = 1.164	0.3811 (NS)	(NS)	0
cryptochrome 2	$F_{5,18}$ = 0.4354	0.8156 (NS)	(NS)	0

NS: non-significant difference ($p > 0.05$).
